# The enhanced-view totally extraperitoneal repair of abdominal bulge after DIEP flap breast reconstruction for breast cancer: a case report

**DOI:** 10.1186/s40792-024-02056-9

**Published:** 2024-11-11

**Authors:** Masami Yako, Yoshiro Imai, Yusuke Suzuki, Kosei Kimura, Mitsuhiro Asakuma, Hideki Tomiyama, Mitsuhiko Iwamoto, Sang-Woong Lee

**Affiliations:** Department of General and Gastroenterological Surgery, Faculty of Medicine, Osaka Medical and Pharmaceutical University, 2-7 Daigaku-machi, Takatsuki, Osaka 569-8686 Japan

**Keywords:** Abdominal bulge, eTEP, DIEP flap, Incisional hernia

## Abstract

**Background:**

The deep inferior epigastric perforator (DIEP) flap for autologous breast reconstruction is associated with higher patient satisfaction and fewer abdominal morbidities at the donor site than the transverse rectus abdominis myocutaneous flap. However, abdominal bulging occurs at a certain frequency, and there is no established treatment. Here, we present a case of laparoscopic hernia repair using the enhanced-view totally extraperitoneal (eTEP) method in a patient with a lower abdominal bulge after DIEP flap reconstruction.

Case presentation.

A 53-year-old woman underwent left nipple-sparing mastectomy, left axillary lymph node dissection, and breast reconstruction with a DIEP flap for left breast cancer 3 years previously. We performed an eTEP method for an abdominal bulge. The absence of a hernia sac facilitated dissection of the retrorectal space, and a left-sided transversus abdominis release was performed, followed by mesh placement. No postoperative abdominal bulging was observed.

**Conclusions:**

Using the eTEP method for repairing an abdominal bulge after DIEP flap reconstruction is advantageous because it facilitates a relatively straightforward dissection of a wide area of the retrorectal space without a hernia sac.

## Background

The deep inferior epigastric perforator (DIEP) flap is commonly used for autologous breast reconstruction because it increases patient satisfaction [1]. The DIEP flap preserves the rectus abdominis muscle and anterior rectus sheath, resulting in reduced morbidity of the donor site hernia and bulge compared with the transverse rectus abdominis myocutaneous (TRAM) flap [2–4]. However, abdominal wall bulging following DIEP flap placement, although infrequent, remains one of the most common morbidities, and there is currently no established method for its probable prevention [5]. Enhanced view totally extraperitoneal (eTEP) repair is a new approach that facilitates extraperitoneal suture closure of defects and wide retrorectal mesh coverage with minimal penetrating fixation, and has recently gained popularity in abdominal hernia surgery [6, 7].

To the best of our knowledge, no other study has reported a case of eTEP repair in a patient with an abdominal bulge after DIEP flap breast reconstruction. Here, we report a case of post-DIEP flap breast reconstruction of an abdominal bulge using eTEP repair.

## Case presentation

**Fig. 1:**
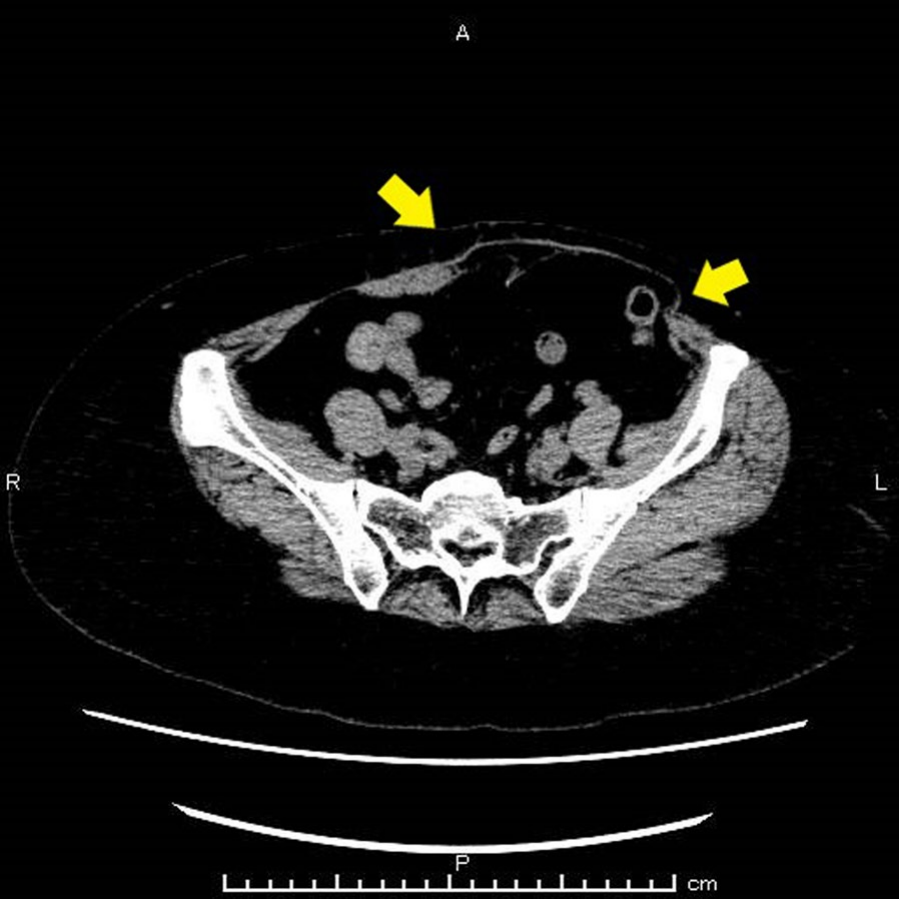
Abdominal computed tomography. Abdominal computed tomography showing a left lower abdominal bulge attributed to atrophy of the left rectus abdominis muscle with no evidence of a fascial defect (yellow arrow)

A 53-year-old Japanese woman presented with left-sided breast cancer. The tumor was diagnosed as T2N1M0 stage IIB (estrogen receptor positive, progesterone receptor negative, and human epidermal growth factor receptor 2 positive). After neoadjuvant chemotherapy, the patient underwent left nipple-sparing mastectomy, left axillary lymph node dissection, and breast reconstruction with a DIEP flap, which is a type of free flap. She had received endocrine therapy for 3 years, and no evidence of breast cancer recurrence was found. However, 3 years postoperatively, she developed extensive distention in the left lower abdomen. Abdominal computed tomography revealed a left lower abdominal bulge attributed to atrophy of the left rectus abdominis muscle, with no evidence of fascial defects. Axial imaging revealed an abdominal bulge 9.5 cm wide (Fig. 1). A diagnosis of abdominal bulge after DIEP flap was made, and we decided to perform eTEP repair.

**Fig. 2:**
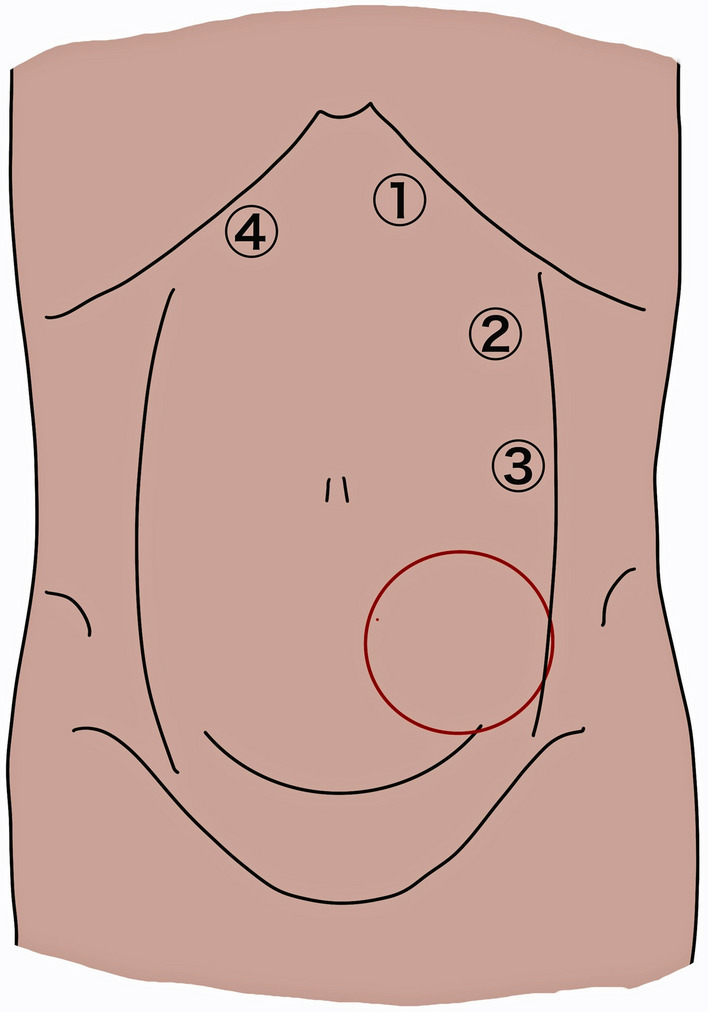
Schema of endoscopic ports placement. The abdominal bulge was located in the left lower abdomen

**Fig. 3:**
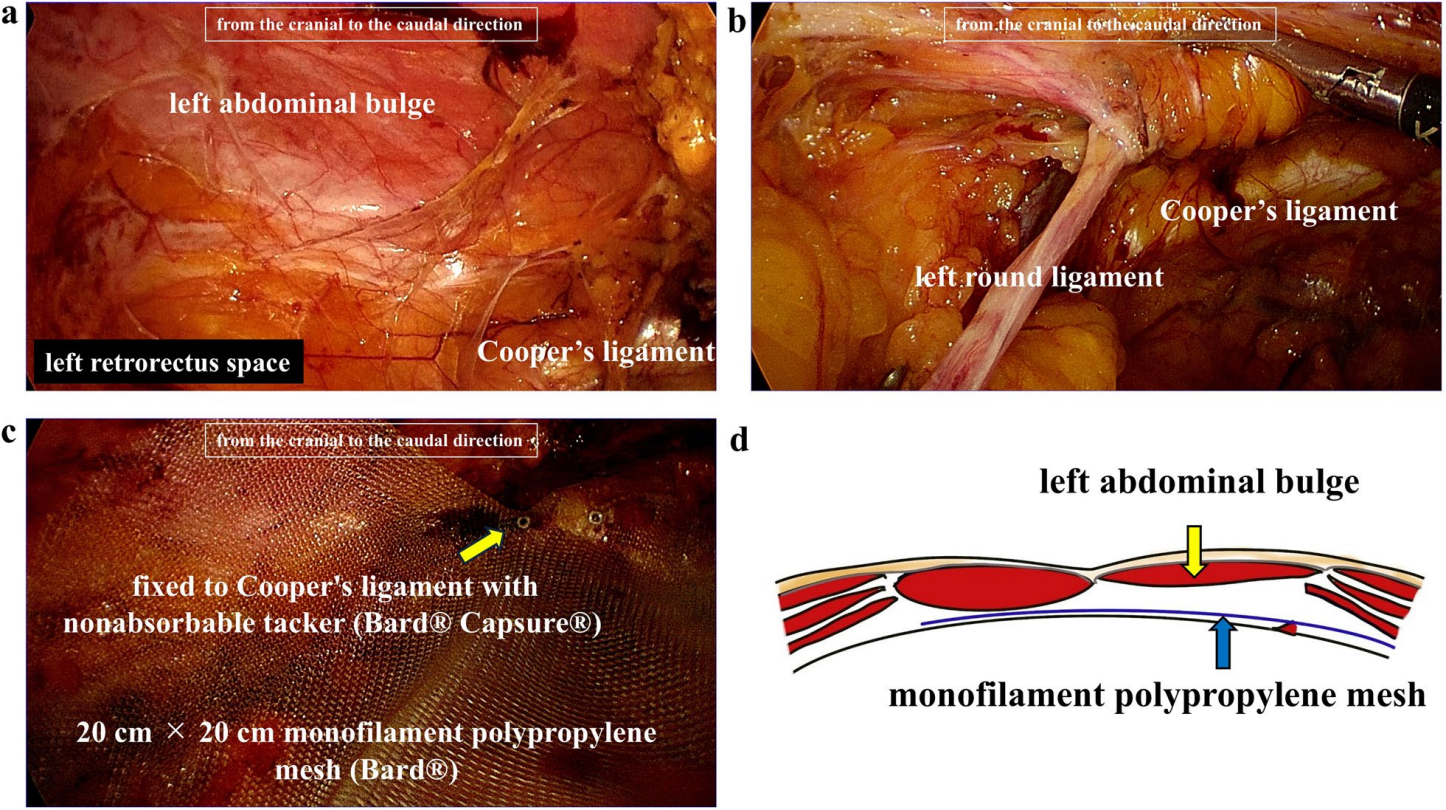
Intraoperative endoscopic view from the cranial to the caudal direction. **a** Dissection of the left retrorectal space in the abdominal bulge was relatively straightforward. **b** The left pubic bone and Cooper’s ligament were exposed, and dissection of the left round ligaments were performed. **c** 20 cm × 20 cm monofilament polypropylene mesh (Bard^®^) was placed in the retrorectal space, fixed to the bilateral Cooper’s ligament with nonabsorbable tacker (Bard^®^ Capsure^®^). **d** Schema of mesh placement in the retrorectal space

The patient was placed in a supine position with both arms tucked at the sides and the lower legs bent under general anesthesia. Figure 2 shows the port placement in this case. A 2-cm skin incision was made at port 1, a 12-mm trocar was inserted into the left retrorectal space using the optical method, and pneumoperitoneum was performed at a pressure of 10 mmHg. The retrorectal space was bluntly dissected, and 5-mm ports were inserted into the Port 2 and Port 3 positions with an endoscopic view. In the upper abdomen, the left posterior rectus sheath was incised through the preperitoneal space, and the right posterior rectus sheath was incised to access the right retrorectal space while preserving the linea alba. A 5-mm port was inserted into port 4. The preperitoneal and bilateral retrorectal spaces were created from the cranial to the caudal direction. Dissection of the retrorectal space in the abdominal bulge was relatively straightforward (Fig. 3a), although some scarring was observed at the deep inferior epigastric perforator sampling site. Although slight pneumoperitoneum occurred during dissection, the peritoneal defect was easily closed using 3–0 absorbable suture. The bilateral pubic bone and Cooper’s ligament were exposed, and the bilateral round ligaments were dissected (Fig. 3b). To provide sufficient space for mesh reinforcement of the abdominal bulge, a transversus abdominis release (TAR) procedure was performed. This involved dissecting the left posterior rectus sheath medial to the neurovascular bundle and dissecting between the transversus abdominis muscle and transverse fascia. Finally, 20 cm × 20 cm monofilament polypropylene mesh (Bard^®^) was placed in the retrorectal space, fixed to the bilateral Cooper’s ligament with nonabsorbable tacker (Bard^®^ Capsure^®^) (Fig. 3c).

Figure 3d shows the mesh placement in this case. The operating time was 298 min, and the blood loss was 5 ml.

**Fig. 4:**
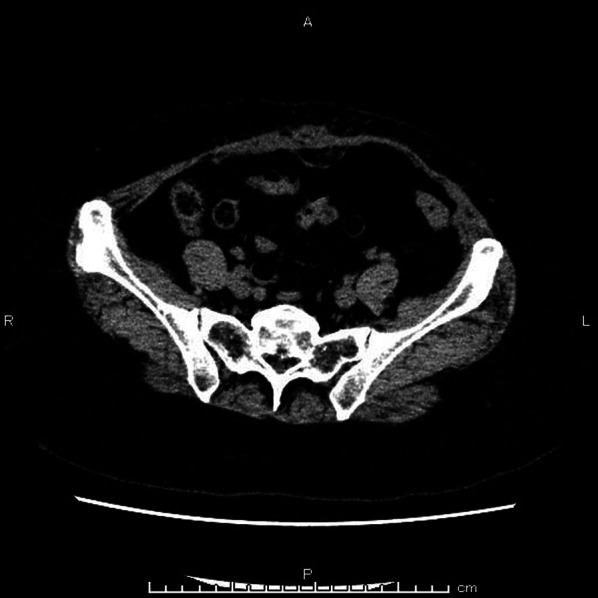
Postoperative abdominal computed tomography. Abdominal computed tomography revealed no abdominal bulge recurrence occurred after 2 months of follow-up

**Fig. 5:**
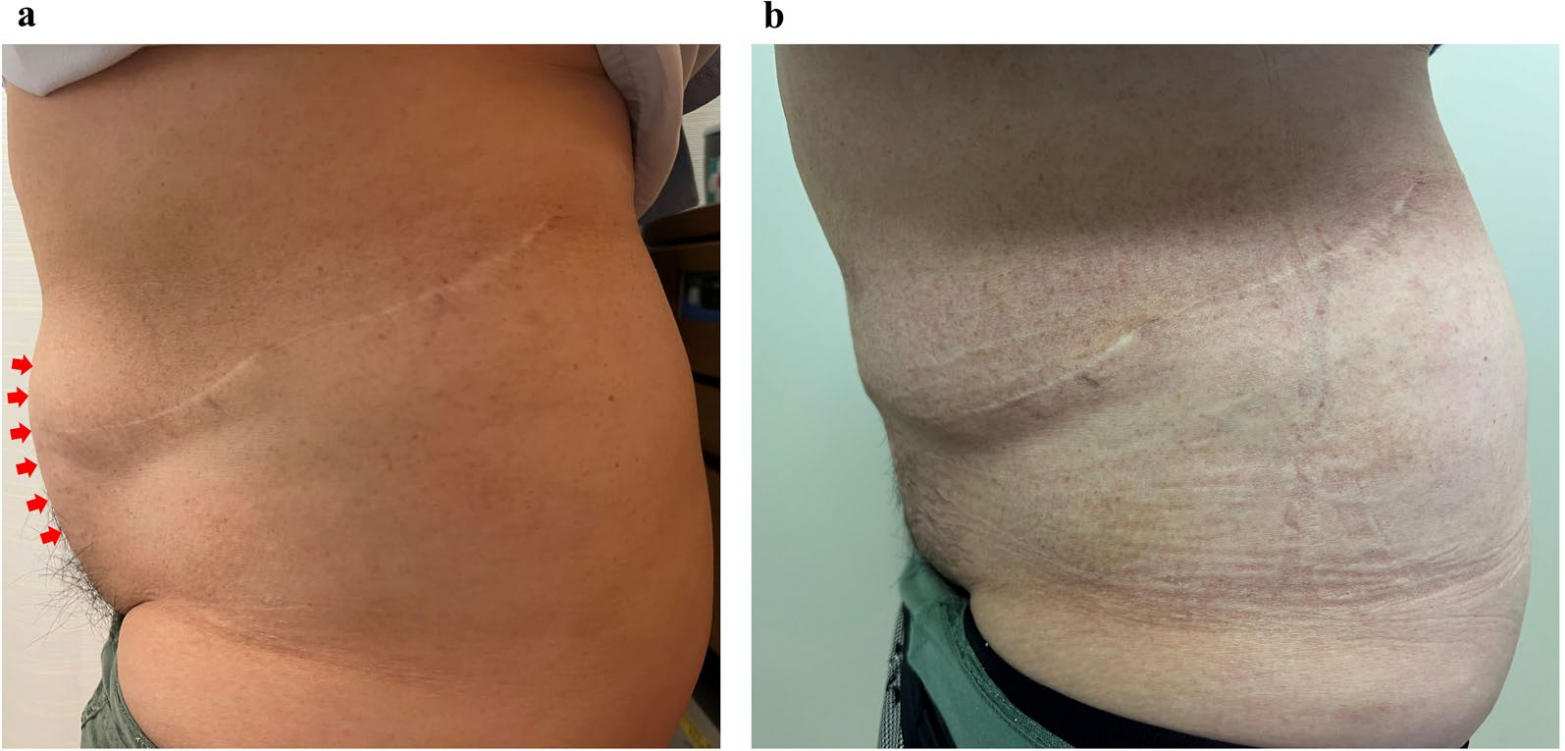
Preoperative and 2-month postoperative lateral views showing that the abdominal bulge had improved. **a** Preoperative lateral view. Red arrows indicate areas of abdominal bulge. **b** Two-month postoperative lateral view

There were no postoperative complications or abdominal bulge recurrences after 2 months of follow-up (Fig. 4). The preoperative (Fig. 5a) and 2-month postoperative (Fig. 5b) lateral views revealed that the abdominal bulge had improved. The patient was satisfied with the clinical outcome.

## Discussion

DIEP flap, which is a free flap, preserves the rectus abdominis muscle and anterior rectus sheath, contributing to reduced donor site morbidity compared to TRAM flap, such as abdominal hernia and bulge [2–4]. However, the frequency of abdominal bulges has been reported to range from 1.6 to 33% [8–13]. An abdominal bulge is defined as abdominal wall laxity without an associated fascial defect [14]. Considering the significance of cosmetic outcomes in breast reconstruction, an abdominal bulge has considerable impact on a patient’s quality of life. Some reports suggest that abdominal bulge may be reduced using a mesh [8, 9, 15], while other studies have indicated no significant effect [3, 10, 11, 16]. Currently, the effectiveness of using a mesh to address abdominal bulges remains inconclusive [5]. Although therapeutic interventions may be required in a few cases, there is currently no established treatment.

The laparoscopic intraperitoneal onlay mesh (IPOM) technique for abdominal hernia has been shown to reduce the risk of wound infection and hospital stay compared with the open technique [17]. However, IPOM causes serious intestine-related complications, such as intestinal adhesion and enterocutaneous fistulas [18]. Furthermore, pain is a problem in both the early and late postoperative periods. It has been suggested that postoperative pain is associated with double crown fixation and transfascial sutures in IPOM plus, with a reported residual chronic pain rate of 12% [19].

The eTEP technique is an adaptation of the Rives–Stoppa technique that involves laparoscopic dissection of the bilateral retrorectal space [6, 7]. Placing the mesh in the retrorectal space prevents direct contact with the intra-abdominal organs, thereby reducing the risk of intestinal complications. Additionally, it obviates the need for Tucker fixation, thereby mitigating the risk of postoperative pain [20].

Currently, there is no established treatment protocol for abdominal bulges after DIEP flap breast reconstruction. While a consensus regarding the efficacy of mesh in preventing abdominal bulging is lacking after DIEP flap breast reconstruction [5], as an abdominal bulge occurs, its use to reinforce the weakened abdominal wall during surgical treatment is considered reasonable. Haddock et al. [16] reported mesh repair for the surgical treatment of an abdominal bulge after DIEP flap breast reconstruction but with mesh placement using onlay repair. However, in abdominal incisional hernia, onlay mesh repair is not recommended because of the high recurrence and infection rates [21]. For recurrence, retrorectal or underlay mesh repair is recommended [22]. Rhemtulla et al. [3] recommended retrorectal mesh repair for abdominal hernia after DIEP flap breast reconstruction. Therefore, we selected the eTEP technique in this case according to abdominal hernia.

Harvesting DIEP flaps for breast reconstruction often involves extensive tissue removal, which may result in a bulky abdominal bulge. Given that the donor site for DIEP flap breast reconstruction is primarily in the lower abdomen, if the IPOM technique is used to reinforce the abdominal bulge, the mesh will extend to the pubic region, which is near the bladder and may cause fixation problems. The eTEP technique is advantageous in this regard as it allows for extensive dissection of the extraperitoneal space, including the bladder. A segment of the retrorectal space was dissected to access the deep inferior epigastric artery; however, most of the rectus abdominis muscle and anterior rectus sheath were not excised, facilitating the ease of dissection of the retrorectal space. Owing to the absence of the need for hernia dissection, the risk of pneumoperitoneum is low. If minor peritoneal injury occurs, it is minimal and easily repaired. Additionally, closure of the hernia defect is unnecessary. This suggests that the risk of interparietal hernia is low owing to the reduced probability of posterior layer breakdown [23]. If the extent of mesh placement is inadequate, TAR should be considered. In cases of a unilateral abdominal bulge, TAR on one side suffices, obviating the need for bilateral TAR. In addition, it is important to consider that the extent of an abdominal bulge can be easily identified as it is observed endoscopically under pneumoperitoneum. With the increasing use of DIEP flap breast reconstruction, the incidence of abdominal bulging is expected to rise. Therefore, we believe that the findings presented in this article are highly relevant and contribute significantly to this field.

## Conclusion

The eTEP repair is safe and effective for treating abdominal bulges after DIEP flap breast reconstruction. Although long-term follow-up is required, we believe that eTEP repair is the most reasonable method for abdominal hernia repair for abdominal bulges after DIEP flap reconstruction.

## Data Availability

Not applicable.

## Abbreviations

DIEP: Deep inferior epigastric perforator
TRAM: Transverse rectus abdominis myocutaneous
eTEP: Enhanced-view totally extraperitoneal
IPOM: Intraperitoneal onlay mesh
TAR: Transversus abdominis release
